# ﻿*Phedimusdaeamensis* (Crassulaceae), a new species from Mt. Daeam in Korea

**DOI:** 10.3897/phytokeys.212.82604

**Published:** 2022-11-03

**Authors:** Tae-Young Choi, Dong Chan Son, Takashi Shiga, Soo-Rang Lee

**Affiliations:** 1 Department of Biology Education, College of Education, Chosun University, Gwangju 61452, Republic of Korea Chosun University Gwangju Republic of Korea; 2 Division of Forest Biodiversity and Herbarium, Korea National Arboretum, Pocheon 11186, Republic of Korea Division of Forest Biodiversity and Herbarium, Korea National Arboretum Pocheon Republic of Korea; 3 Faculty of Education, Niigata University, Niigata, Japan Niigata University Niigata Japan

**Keywords:** Molecular diagnosis, new species, *Phedimus*, phylogeny

## Abstract

*Phedimus* individuals from Mt. Daeam, once referred to as *Phedimussikokianus*, exhibit certain morphological characters that are unique within the genus. *Phedimus* is one of the most notorious groups for taxonomic problems due to the high morphological variation found in leaf shape, stem numbers, phyllotaxis and seed structure. Taxa in *Phedimus* also easily hybridize, further leading to taxonomic confusion. To carefully confirm the identity of the putative new species from Mt. Daeam, we examined morphological characters from ~100 herbarium sheets of six closely related *Phedimus* species. A molecular phylogenetic approach was also employed to delimit the species boundary and infer the phylogenetic relationships among the seven *Phedimus* species, including the species from Mt. Daeam. Both morphological and molecular phylogenetic results indicated that the species found on Mt. Daeam is a new species that is more closely related to *P.middendorffianus* and *P.takesimensis* than to the remaining four *Phedimus* species. Here, we provided a full description of the new species *P.daeamensis* as well as an updated key for the seven *Phedimus* species examined.

## ﻿Introduction

Until ‘t Hart (1995) resurrected the genus *Phedimus* Rafinesque (Rafinesque 1817) by separating it from *Sedum*, the taxonomic group had been buried for approximately over a century. Since its resurrection, ca. 20 species have been added to *Phedimus* (Fu et al. 2001; ‘t Hart and Bleij 2003). Most taxa in the genus are distributed throughout Eurasia; their primary habitats are rocky slopes and grasslands (Fu et al. 2001). It is now widely accepted that the two genera, *Phedimus* and *Sedum*, are primarily distinguished by their leaf and testa shapes, which is further supported by several molecular studies (Ohba et al. 2000; Mayuzumi and Ohba 2004; Gontcharova et al. 2006; Gontcharova and Gontcharov 2009). *Phedimus*, a perennial herb, is divided into two subgenera (*Phedimus* and *Aizoon*) that differ in petal colors, sterile stems, and testa structures. In East Asia, approximately 15 taxa are recognized based on the aforementioned morphological traits, with more emphasis on the number of stems and phyllotaxis (Borisova 1939; Fu et al. 2001; Ohba 2001). However, in many cases, the delimitation of taxa is challenging because of the extensive morphological variations (Mayuzumi and Ohba 2004; Moon and Jang 2020) within the genus. Furthermore, the wide use of *Phedimus* as a core source of horticultural cultivars complicates the taxonomic issues (Stephenson and Harris 1991; Han et al. 2020). Given the taxonomic challenges, reporting a new species only by morphological features (e.g., Chao 2020) may need an additional molecular examination.

There are eight *Phedimus* species including two endemic species and one with two infraspecific taxa in Korea [Phedimusaizoon(L.)‘t Hartvar.aizoon, P.aizoon(L.)‘t Hartvar.latifolius (Maxim.) H. Ohba, *P.kamtschaticus* (Fisch. & C.A. Mey.) ‘t Hart, *P.latiovalifolius* (Y.N. Lee) D.C. Son & H.J. Kim, *P.middendorffianus* (Maxim.) ‘t Hart, *P.selskianus* (Regel & Maack) ‘t Hart, *P.takesimensis* (Nakai) ‘t Hart, *P.zokuriensis* (Nakai) ‘t Hart] (Park 2007; Korea National Arboretum 2021). According to Lee et al. (2003), all Korean species belong to the subgenus Aizoon. However, some species show considerable intraspecific morphological variation leading to taxonomic confusion, particularly where identity and species boundaries are concerned (Ryu et al. 2011; Moon and Jang 2020). *Phedimuskamtschaticus* (Fisch.) ‘t Hart is a compelling example of the marked infraspecific morphological variation (e.g., wide variety of leaf shapes) (Park 2007; Moon and Jang 2020). In fact, during a 2019 study of specimens at the herbarium of the Korea National Arboretum (KH), multiple sheets collected on Mt. Daeam and Gangwon province differed substantially from the rest of the collection. Specimens with unique morphotypes were identified as *P.kamtschaticus* or *P.middendorffianus* (Maxim.) t’ Hart. (Oh 1985; Oh et al. 2015). Of those, the Mt. Daeam specimens were identified as *P.sikokianus* (Chung and Kim 1989); however, the distribution of this species is restricted to high mountain areas in Japan, suggesting that the Mt. Daeam specimens were likely misidentified. Accordingly, a close investigation of the *Phedimus* plants collected on Mt. Daeam was carried out.

Mt. Daeam, is a high-altitude mountain (> 1300 m) in Korea, which owing to its diverse geographical and environmental characteristics is an area of substantial biodiversity (Ministry of Environment 2007). The primary soil components of Mt. Daeam are granite and gneiss followed by sand (~11%), silt, and clay (~10%; Ministry of Environment 2007). Notably, Korea’s only reported peatland (Min et al. 2000; Kim et al. 2005), Yongneup, which consists of five swamps, is located in high altitudes (1000–1200 m) of the mountain. The climate is typically temperate with cold and humid conditions (average annual temperature = ~10 °C and average annual relative humidity = 71%; Ministry of Environment 2007), thus serving as a refuge for several northern plants (Min et al. 2000; Kim et al. 2005). Over 300 taxa, including 20 Korean endemics, have been recorded on Mt. Daeam, and ca. 70 are protected by Korean law (Ministry of Environment 2007). The unique environmental properties of Mt. Daeam may have contributed to high species richness as discoveries of new plant taxa are ongoing (Lee et al. 2013; Gil et al. 2019).

In the present study, we report a new plant species, *P.daeamensis* T.Y. Choi & D.C. Son of the genus PhedimussubgenusAizoon. We described the morphological characters and habitat features of the new species with a detailed botanical illustration in gray-scale hand drawing. To delimit the species boundary from the six closest related taxa, we performed morphological observations as well as a molecular phylogenetic study. A key to the Korean species of Phedimus (subgenus Aizoon) including the new species was established based on the examined morphological characters.

## ﻿Materials and methods

### ﻿Morphological examination

**Figure 1.**
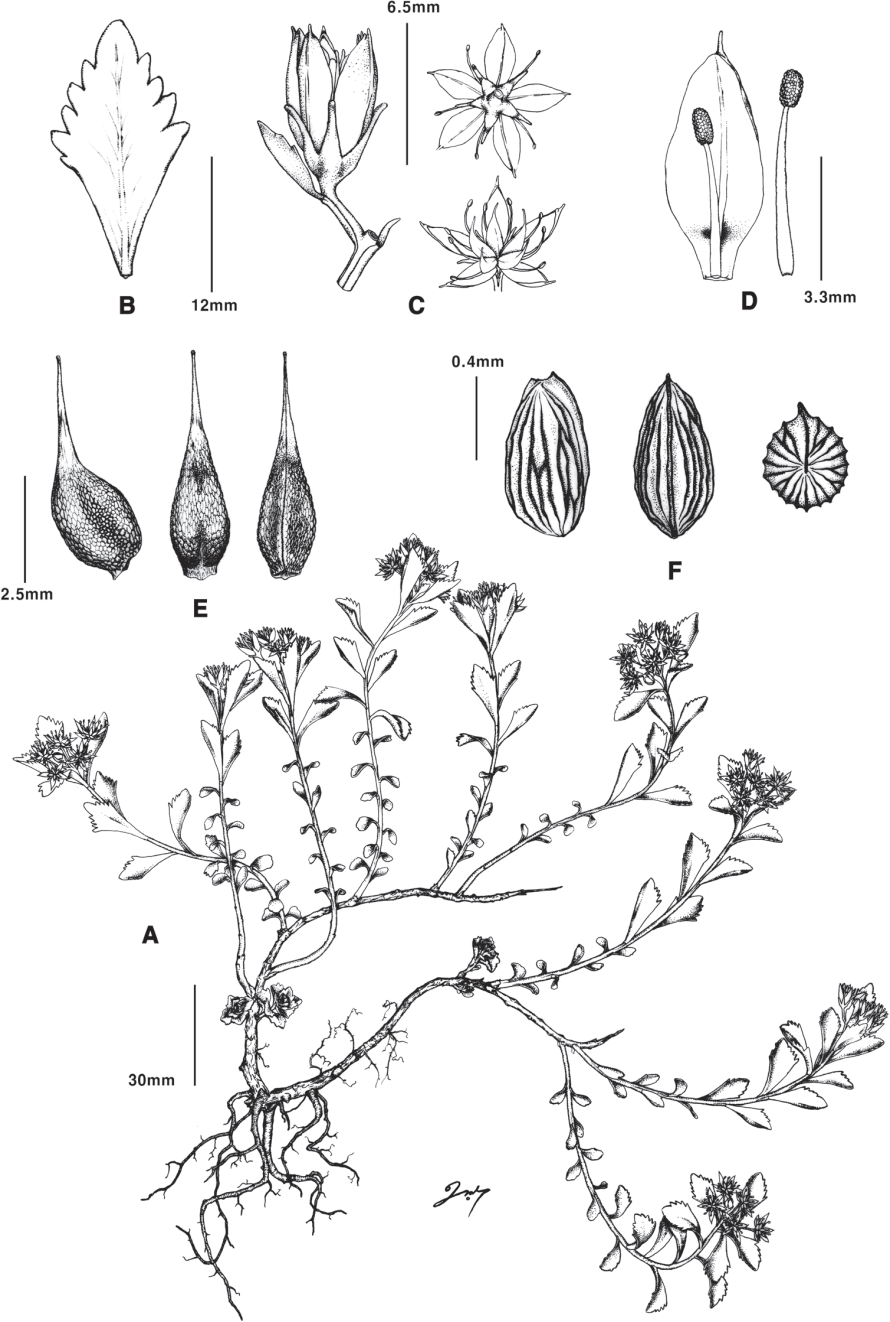
*Phedimusdaeamensis***A** habit **B** leaf **C** flower **D** petal and stamen **E** carpel **F** seed. (Illustrated by Kyungsoo Eo).

We collected four living samples of *P.daeamensis* and prepared a voucher specimen. Referring to the relevant protologues, floras, and monographs (Fu et al. 2001; Ohba 2001; Lee et al. 2003; Park 2007), we determined six target congeneric taxa for examination. All samples used for the study were collected legally. To compare the morphological characteristics of the new species with the six most closely related congeners, we borrowed ca. 100 herbarium specimens deposited in the KH and the Makino Herbarium (Suppl. material 1: Table S1). Using an Olympus dissecting stereo microscope (SZX16), morphological observations were made on all parts of the plants with a particular focus on the shape of leaves and leaf parts as well as the features of the reproductive organs. Microscopic floral parts such as the carpels and stamens were dissected when required. Five characters associated with the leaf (the phyllotaxis, length and width of the leaves, shape of the petioles, and the blades), and several associated with the flower (including size and shape of the calyx lobes, number and shape of the petals, and numbers of stamen and carpel; Table 1 and Suppl. material 1: Table S2; Fig. 1) were assessed.

**Table 1.** Summary of diagnostic characters observed in *Phedimusdaeamensis* and the two morphologically closest taxa. The full diagnostic morphological characters of all seven *Phedimus* taxa investigated in the study are presented as supplementary information (Suppl. material 1: Table S2).

	*P.middendorffianus*	*P.sikokianus*	*P.daeamensis*
Leaves	alternate	opposite	alternate
blade shape	linear-spatulate	widely oblanceolate to obovate	obovate
blade size	1.2–4 cm long, 0.2–0.5 cm wide	0.8–2.3 cm long, 0.6–1.3 cm wide	1–2.3 cm long, 0.5–1.2 cm wide
margins	margin apically serrate 2–3, apex obtuse	margin apically to mid crenate 2–4, apex rounded	margin apically to mid serrate 4–5, apex obtuse
Calyx lobes	5, linear, 2–3 mm long, apex obtuse	5, lanceolate, 2–3 mm long, apex obtuse	5, lanceolate, 3–4 mm long, apex obtuse
Seeds	obovoid	ellipsoid, ca. 0.8–1 mm long	obovoid, ca. 0.7–1 mm long

### ﻿Molecular diagnosis

To delimit the new species from the six most closely related taxa we examined their phylogeny. Sixteen samples of the seven taxa (three *P.daeamensis* and remaining of the six closely relatives) were collected from 14 localities across South Korea and Japan (see Suppl. material 1: Table S3). Three samples of the new species were included to determine the species’ monophyly. We first examined the three regions of cpDNA (*atp*F-*atp*H IGS, *trn*L-*trn*F IGS, and psbA-*trn*H IGS) and the nrITS region discovered by Mayuzumi and Ohba (2004) in test samples from all seven taxa. After the DNA polymorphism test, we excluded the *atp*F-*atp*H and *trn*L-*trn*F IGS regions because of the lack of polymorphism among the seven taxa. Genomic DNAs of the 16 samples were extracted from either fresh or dried leaf samples using DNeasy plant mini kit (Qiagen, Hilden, Germany) following the manufacturer’s protocol. The PCR amplifications were carried out using GeneAmp PCR system 9700 with a total reaction volume of 50 uL containing 50 ng of template DNA. The amplification conditions are provided in Suppl. material 1: Table S4. After a series of purification steps performed by Macrogen (Seoul, Korea), the PCR products were sequenced on an ABI Prism 3730XL genetic analyzer (Applied Biosystems, Waltham, USA) using ABI Prism BigDye terminator v 3.1 cycle sequencing kit (Applied Biosystems, Waltham, USA) at the Macrogen facility (Macrogen, Seoul, Korea).

We also included seven accessions of three *Phedimus* taxa (*P.latiovalifolius*, P.aizoonvar.floribundus, *P.takesimensis*) downloaded from GenBank to test the species boundaries across all *Phedimus* taxa co-occurring in Korea and Japan (Suppl. material 1: Table S5). We assigned two *Rhodiola* species (*Rhodiolabrevipetiolata* and *R.alsia*) to the out-group based on previous phylogenetic research (Mayuzumi and Ohba 2004). All sequences were edited and aligned using Geneious Aligner in Geneious Prime ver. 2020.0.5, whereas other parameters were set as defaults. We then manually adjusted the aligned sequences. All DNA sequences obtained from the study were deposited in GenBank (accession numbers in Suppl. material 1: Table S3). We inferred phylogeny for the nrITS and cpDNA regions independently. Data concatenation was not considered because previous studies on *Phedimus* phylogeny showed substantial incongruence between nrITS and cpDNA trees (Seo et al. 2020). The phylogenetic trees were instead inferred from maximum likelihood (ML) and Bayesian interference (BI) methods. ML analyses were performed using RAxML plugin v4.0 implemented in Geneious Prime with the GTR CAT approximation (Lartillot and Philippe 2004). Node supports were evaluated with 1000 bootstrap replicates (Felsenstein 1985). BI analyses were performed in MrBayes 3.2.6 (Ronquist and Huelsenbeck 2003) using four chains (three heated and one cold) for 5 million generations while sampling every 1000^th^ generation. The first 25% of the samples were discarded as a burn-in, and the remaining trees were used to produce a 50% majority-rule consensus tree.

## ﻿Results

### ﻿Morphological examination

We used the plant habit, leaf shapes, and margins to distinguish the newly described *Phedimus* species (Fu et al. 2001; Ohba 2001; Lee et al. 2003; Park 2007). *Phedimusaizoon*, *P.kamtschaticus*, and *P.takesimensis* were easily distinguished from the five remaining species by height (> 20 cm) and leaf margin (entirely toothed; Table 1). *Phedimusdaeamensis* was morphologically most similar to *P.middendorffianus* and *P.sikokianus* in terms of the following characters: fibrous roots, not robust, and stems shorter than 20 cm, somewhat prostrate (Table 1). However, *P.daeamensis* was distinguished from *P.middendorffianus* by its leaf shape (*P.daeamensis* leaf shape-obovate, 1–2.3 cm long; leaf margins with 4–5 teeth from apex to mid; sepals (calyx lobes) lanceolate) and from *P.sikokianus* by its leaf phyllotaxis and seed shape (Fig. 1 and Table 1).

### ﻿Taxonomic treatment

#### ﻿ Phedimus daeamensis

Taxon classificationPlantaeSaxifragalesCrassulaceae

T.Y. Choi & D.C. Son sp. nov.

847ECD52-F5D1-5BFC-A9B0-B6E5E0B5B92A

urn:lsid:ipni.org:names:77307628-1

Fig. 1

##### Type.

Republic of Korea. Gangwon-do, Inje-gun, Buk-myeon, Wolhak-ri, Mt. Daeam. Elevation 1,000 m. 20 August 2014. K.H. Lee & S.K. So 0001 (holotype KH; isotypes 2 sheet, KH).

##### Perennial herbs.

Rhizome woody, elongated. Roots not tuberous; rootstock not robust. Stems numerous, more basally branched, tufted, creeping, ascending, 12–21 cm long, glabrous. Leaves alternate, sessile, coarsely arranged; leaf blade obovate, 1–2.3 cm long, 0.5–1.2 cm wide, flat, base narrowly cuneate, margin apically to mid serrate 4–5×, entire at base, apex obtuse; lower leaves almost all entire. Inflorescence corymbiform-cymose, many-flowered; bracts leaf-like. Flowers bisexual, mostly 5-merous, shortly pedicelled. Calyx tube 2.1–3.2 mm long; lobes spurless, lanceolate, 1–1.2 mm long, apex obtuse. Petals free, yellow, lanceolate to oblong, 5–6.5 mm long, abaxially keeled, apex acuminate, spreading at anthesis. Stamens 10, in 2 series, erect, shorter than petals, those opposite to petals adnate to them to 1/4 of length from the base; anthers red, ellipsoid, ca. 1 mm long; filaments yellow. Pistils 4.5–5 mm long; ovaries ca. 2.5 mm long, connate at the base; styles slender, 2–3 mm long. Carpels 5, erect, equaling or slightly shorter than the petals, adaxially gibbous, shortly connate at the base. Follicles greenish, stellately and horizontally spreading, ca. 4 mm long, with a very short beak. Seeds 0.8–0.9 mm long, brown, obovoid, scalariform, ribbed, striate.

Flowers in May to June, fruiting in July to August.

##### Distribution and habitat.

Republic of Korea (Prov. Gangwon). Stony cliffs and rock crevices, at ca. 1000 m.

##### Etymology.

The specific epithet, “***daeamensis***”, is based on the name of the location, Mt. Daeam, where *Phedimusdaeamensis* was discovered.

##### Korean name.

Dae-am-gi-rin-cho.

##### Molecular diagnosis.

In total, 32 sequences of two DNA regions (ITS and *psb*A-*trn*H IGS) were newly obtained from the 16 accessions of *P.daeamensis* and the six most closely related taxa (Suppl. material 1: Table S3). We also used 15 sequences from eight accessions obtained from GenBank (P.aizoonvar.floribundus, *P.latiovalifolius*, *P.takesimense*) for the phylogenetic analysis. The lengths of the ITS and *psb*A-*trn*H IGS alignment were 588 and 272 base pairs, respectively (Table 2). After an alignment of 24 accessions, we found 173 variable sites and 144 of these were parsimony informative (Table 2 and Suppl. material 1: Table S6). Overall, the GC ratio was 50.5% and 22.5% for ITS and *psb*A-*trn*H IGS, respectively (Table 2). K2P genetic distances among in-group individuals ranged from 0 to 0.043 (mean 0.023) for ITS and 0 to 0.048 (mean 0.018) for *psb*A-*trn*H IGS (Table 2). We also found a 6 bp inversion in the *psb*A-*trn*H IGS of all *P.daeamensis* accessions and one accession of *P.takesimensis* (Suppl. material 1: Table S6). We excluded this inversion from further phylogenetic analysis.

**Table 2.** Results of the cpDNA data sets used in this study. The out-group taxa were included in the analyses, except for the K2P distance.

	ITS	*psbA-trn*H IGS
Sequence length (bp)	572–579	234–266
Aligned length (bp)	588	272
Mean G+C ratio (%)	50.5	22.5
No. of variable characters	144	29
No. of parsimony informative characters (%)	120 (85.7)	24 (82.8)
K2P distance (mean)^*^	0–0.043 (0.023)	0–0.048 (0.018)

* Out-group taxa excluded.

**Figure 2.**
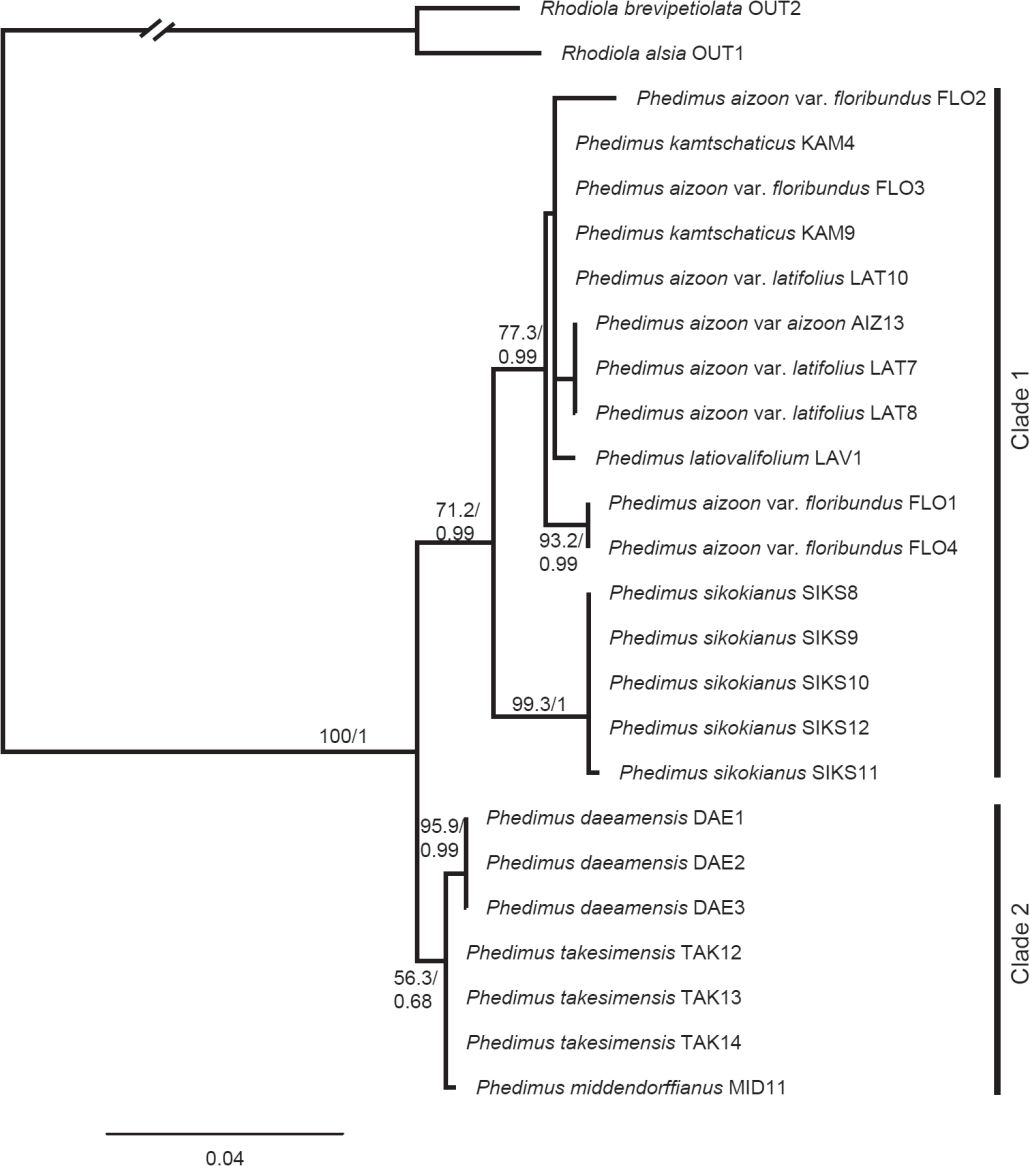
Maximum likelihood tree for individuals of *Phedimusdaeamensis* and related taxa based on nrITS. Numbers above branches indicate bootstrap values (> 50%) and posterior probabilities (> 0.5).

**Figure 3.**
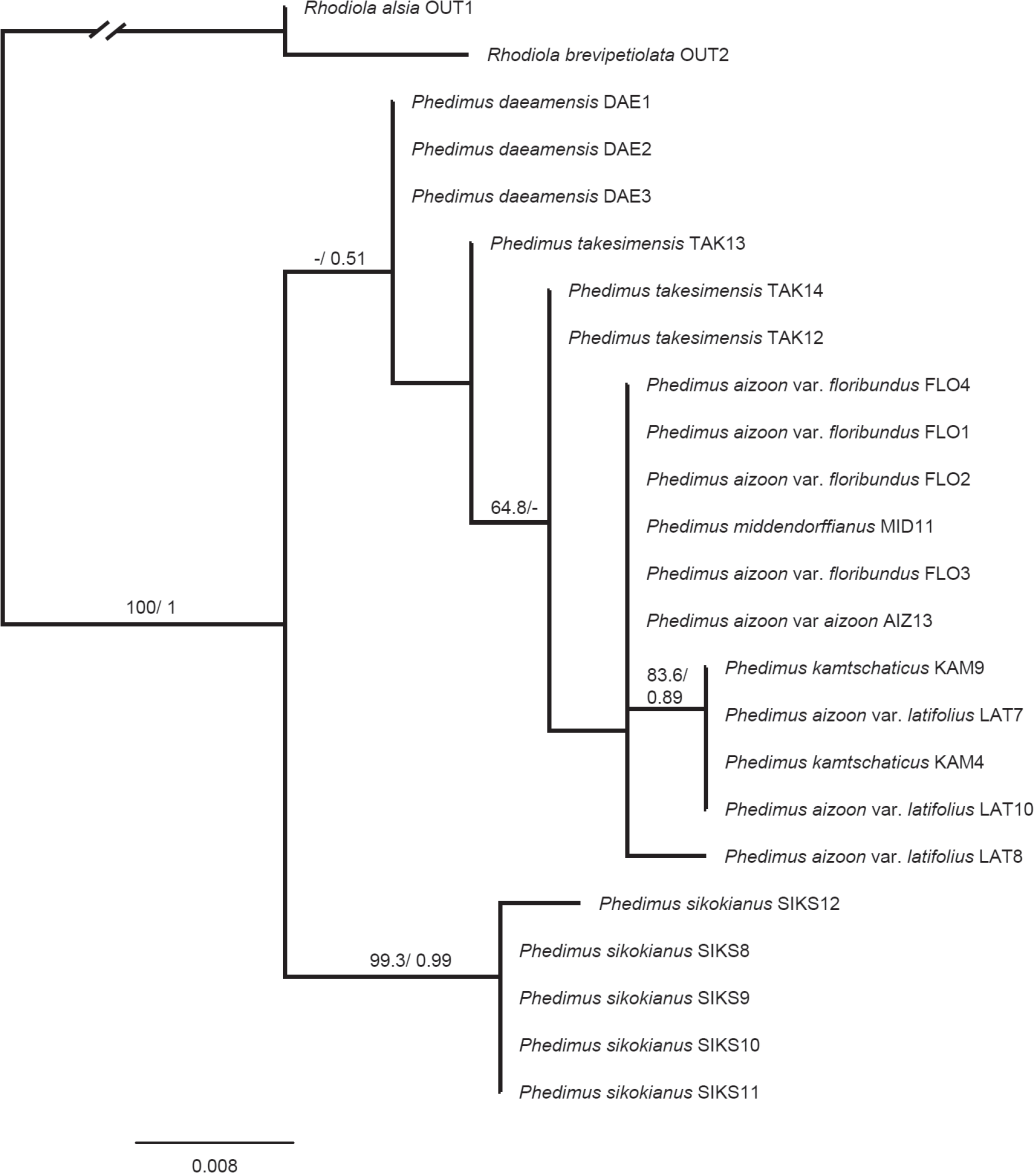
Maximum likelihood tree for individuals of *Phedimusdaeamensis* and related taxa based on *psb*A-*trn*H IGS. Numbers above branches indicate bootstrap values (> 50%) and posterior probabilities (> 0.5).

Overall, the inferred phylogenies from the two regions differ, particularly in the basal nodes (Figs 2, 3). There was a congruence between the ML and BI trees inferred from the ITS and *psb*A-*trn*H IGS data sets (Figs 2, 3, Suppl. materials 2, 3: Figs S1, S2; posterior probabilities are indicated in ML trees). In the *psb*A-*trn*H IGS trees, *P.daeamensis* was separated but formed an unresolved polytomy (Fig. 3 and Suppl. material 3: Fig. S2). *Phedimussikokianus* formed a monophyletic group, whereas all other species showed more complicated and mixed clustering patterns (Fig. 3 and Suppl. material 3: Fig. S2). In the ITS trees, two major clades were recognized, but only clade 1 was statistically robust (Fig. 2 and Suppl. material 2: Fig. S1). The three samples of the putative new species, *P.daeamensis*, formed a well-supported monophyletic clade (bootstrap value; BS = 95%; posterior priority; PP = 0.99) that was separated from the other species. *Phedimusdaeamensis* again formed a clade together with *P.middendorffianus* (one sample) and *P.takesimensis* (three samples), but the statistical support was very weak (Fig. 2 and Suppl. material 2: Fig. S1). All accessions of *P.sikokianus* formed a well-supported clade (BS = 95.9%; PP = 0.99) with samples of *P.kamtschaticus* and *P.aizoon*, both of which were not monophyletic (Fig. 2, Suppl. material 2: Fig. S1). *Phedimuslatiovalifolius* was nested within a clade containing samples of *P.kamtschaticum* and *P.aizoon* (Fig. 2).

### ﻿Key to *Phedimusdaeamensis* and related species

**Table:** 

1	Stems 1–3, erect; leaves lanceolate, apex acuminate	***Phedimusaizoon***
–	Stems many, ascending to prostrate; leaves spathulate, obovate, oblanceolate or elliptic-oblanceolate, apex obtuse to rounded	**2**
2	Roots thick, robust; stems 20–50 cm long, ascending	**3**
–	Roots fibrous; stems less than 20 cm long, prostrate	**4**
3	Leaves oblanceolate or spathulate, margins serrate in upper half	***Phedimustakesimensis***
–	Leaves spathulate, obovate or elliptic, margins entire or with few acute to obtuse teeth	***Phedimuskamtschaticus***
4	Leaves broadly ovate, margins irregularly dentate	***Phedimuslatiovalifolius***
–	Leaves obovate to linear, margins serrate or crenate	**5**
5	Leaves obovate, somewhat concave	**6**
–	Leaves linear-spathulate or elliptic-oblanceolate, flat	**7**
6	Leaves opposite, margins crenate, seeds ellipsoid	***Phedimussikokianus***
–	Leaves alternate, margins serrate, seeds obovoid	***Phedimusdaeamensis***
7	Stems prostrate; leaves 1.2–2.5 cm × 3–5 mm, with 2 or 3 teeth	***Phedimusmiddendorffianus***
–	Stems decumbent; leaves 2.5–3.5 cm × 1.1–1.6 cm, with many teeth	***Phedimuszokuriensis***

## ﻿Discussion

*Phedimus* has been a rather unexplored taxonomic group until the resurrection of the genus by ‘t Hart (1995). Since then, the genus has attracted substantial attention because of its frequent use in horticultural practices (Han et al. 2020). However, *Phedimus* is difficult to categorize taxonomically because of complex morphological variations, potential hybridization, and introgression among congeneric taxa (Yoo and Park 2016; Han et al. 2020). The possibility of polyploidy (including aneuploidy in *Phedimus*) was also suggested by several empirical studies (Baldwin 1943; Uhl and Moran 1972; Amano 1990; Amano and Ohba 1992; Chung et al. 2020). Accordingly, taxon delimitation in the genus *Phedimus* based solely on morphological characters can easily be misleading and inconclusive, particularly in the early developmental stages when there are no well-developed reproductive organs present. With the recent advancement of molecular tools, molecular markers have helped overcome many of the limitations associated with species delimitation (Pelser et al. 2017; Perkins 2019). Coupled with morphological examinations, our molecular analysis found that *P.daeamensis* is well-separated from the *P.kamtschaticus* complex and *P.sikokianus*, although the taxa were nearly indistinguishable by morphological characters in the early developmental stages.

Overall, our study characterized the morphological distinctiveness of the newly described species (*P.daeamensis*) from the six closest related congeners. However, most characters of examination were vegetative and thus showed significant infraspecific variation across varying environments. *Phedimuskamtschaticus* and *P.aizoon* showed substantial morphological variation. Although *P.kamtschaticus*, the most commonly occurring *Phedimus* species in Korea (Korea National Arboretum 2016), can easily be distinguished from the newly described species (*P.daeamensis*) when the plants are fully mature, the identification may not be as straightforward in the early stage of the development. Our results highlighted a key morphological feature differentiating *P.daeamensis* from *P.kamtschaticus*; however, extreme care must be taken with juvenile plants. *Phedimusdaeamensis* was initially recognized as *P.sikokianus* by Chung and Kim (1989) because of its morphological affinities. According to our results, the putative new species differs from *P.sikokianus* by the type of phyllotaxis and the seed shape, but infraspecific variations in those characters should be considered. The leaf shape of *P.middendorffianus* was prominently linear, which differs substantially from the remaining congeners; however, a very limited number of specimens were examined in our study (Suppl. material 1: Table S1). The morphological analyses we performed provided several key characters distinguishing *P.daeamensis* from the remaining six *Phedimus* taxa, however, some taxa, e.g., *P.middendorffianus* and *P.sikokianus*, only had a limited number of sheets. Therefore, we further employed a molecular phylogenetic approach to support the morphological results.

Notably, phylogenetic trees reconstructed based on the ITS and *psb*A-*trn*H IGS regions were consistent with the morphological results. In both ML trees from nrITS and cpDNA, the three morphotypes sharing the same morphological characters as the newly reported *P.daeamensis* came out as a monophyletic group or as an unresolved polytomy. *Phedimusdaeamensis* was always placed separate from both *P.kamtschaticus* and *P.sikokianus*, but the phylogenetic relationship of the species with its closest related taxa was inconclusive because of low clade support and inconsistency between nrITS and cpDNA trees. In the ITS trees, *P.daeamensis* fell into the same clade as *P.takesimensis* and *P.middendorffianus* (Fig. 2), whereas in the *psb*A-*trn*H IGS trees, *P.daeamensis* was “sister” to all other species except for *P.sikokianus* (Fig. 3). Although the phylogenetic relationship among *P.daeamensis*, *P.takesimensis*, and *P.middendorffianus* was rather ambiguous, the taxa were relatively easy to distinguish based on morphological characters. *Phedimustakesimensis* was much larger (20–50 cm tall) and characterized by thick roots, whereas *P.middendorffianus* has linear leaves. Considering all the evidence and consistent with our hypothesis, *P.daeamensis* is a species in its own right and well-separated from the remaining six species.

## Supplementary Material

XML Treatment for Phedimus daeamensis

## Supplementary materials

Supplementary material 1 Tables S1–S6 Data type occurence, morphology (docx./xslx. files in zip. archive) Explanation note **Table S1.** List of examined specimens for morphological study. **Table S2.** Diagnostic characters observed in *Phedimusdaeamensis* and the six closest related taxa. **Table S3.** Voucher information and GenBank accession numbers for the 16 accessions of *P.daeamensis* and closely related taxa examined in this study. **Table S4.** PCR/sequencing primers and PCR cycling conditions for the DNA regions examined in this study. **Table S5.** Voucher information and GenBank accession numbers for 10 *Phedimus* accessions downloaded from GenBank. **Table S6.** Variable sites obtained from the ITS and *psb*A-*trn*H IGS regions. https://binary.pensoft.net/file/765262This dataset is made available under the Open Database License (http://opendatacommons.org/licenses/odbl/1.0/). The Open Database License (ODbL) is a license agreement intended to allow users to freely share, modify, and use this Dataset while maintaining this same freedom for others, provided that the original source and author(s) are credited.Tae-Young Choi, Dong Chan Son, Takashi Shiga, Soo-Rang Lee

Supplementary material 2 Figure S1 Data type Image (Adobe PDF file) Explanation note **Figure S1.** Bayesian inference tree for individuals of *P.daeamensis* and related taxa based on ITS. Numbers above branches are posterior probabilities. https://binary.pensoft.net/file/765263This dataset is made available under the Open Database License (http://opendatacommons.org/licenses/odbl/1.0/). The Open Database License (ODbL) is a license agreement intended to allow users to freely share, modify, and use this Dataset while maintaining this same freedom for others, provided that the original source and author(s) are credited.Tae-Young Choi, Dong Chan Son, Takashi Shiga, Soo-Rang Lee

Supplementary material 3 Figure S2 Data type Image (Adobe PDF file) Explanation note **Figure S2.** Bayesian inference tree for individuals of *P.daeamensis* and related taxa based on *psb*A-*trn*H IGS. Numbers above branches are posterior probabilities. https://binary.pensoft.net/file/765264This dataset is made available under the Open Database License (http://opendatacommons.org/licenses/odbl/1.0/). The Open Database License (ODbL) is a license agreement intended to allow users to freely share, modify, and use this Dataset while maintaining this same freedom for others, provided that the original source and author(s) are credited.Tae-Young Choi, Dong Chan Son, Takashi Shiga, Soo-Rang Lee
