# Supplementary material for: ﻿Phedimusdaeamensis (Crassulaceae), a new species from Mt. Daeam in Korea
Source: PhytoKeys. 2022 Nov 3;212:57–71. doi: 10.3897/phytokeys.212.82604 (PMC9836587; doi:10.3897/phytokeys.212.82604)
Supplement: Supplementary material 1 — Tables S1–S6 [file phytokeys-212-057_article-82604__-s001.zip › 82604_1C-1-A_revised_Table_S4 SM-1.docx]

Table S4. PCR/sequencing primers and PCR cycling conditions for DNA regions examined in this study. Primer names follow the original publications.

|  | PCR/sequencing primers | | PCR cycling condition (35 cycle) | | | | |
| --- | --- | --- | --- | --- | --- | --- | --- |
|  | Forward primer | Reverse primer | Pre-denaturation (95°C) | Denaturation (95°C) | Annealing (54°C) | Extension (72°C) | Final extensions (72°C) |
| nrITS | ITS1^1^ | ITS4^1^ | 3 min | 1 min | 30 sec | 1.5 min | 7 min |
| *psb*A-*trn*H IGS | psbA^2^ | trnH^2^ | 3 min | 1 min | 30 sec | 40 sec | 7 min |

1. White TJ, Bruns T, Lee S, Taylor J (1990) Amplification and direct sequencing of fungal ribosomal RNA genes for phylogenetics. PCR Protocols: A Guide To Methods And Applications 18: 315-322.

2. Sang T, Crawford DJ, Stuessy TF (1997) Chloroplast DNA phylogeny, reticulate evolution, and biogeography of *Paeonia* (Paeoniaceae). American Journal of Botany 84: 1120–1136.
