# Supplementary material for: ﻿Phedimusdaeamensis (Crassulaceae), a new species from Mt. Daeam in Korea
Source: PhytoKeys. 2022 Nov 3;212:57–71. doi: 10.3897/phytokeys.212.82604 (PMC9836587; doi:10.3897/phytokeys.212.82604)
Supplement: Supplementary material 1 — Tables S1–S6 [file phytokeys-212-057_article-82604__-s001.zip › 82604_1C-1-A_revised_Table_S5 SM-1.docx]

Table S5. Voucher information and GenBank accession numbers for 10 *Phedimus* accessions downloaded from GenBank.

| Accession | | Locality | GenBank accession  number | |
| --- | --- | --- | --- | --- |
|  |  |  | ITS | *psb*A-*trn*H IGS |
| FLO1 | *Phedimus aizoon* var. *floribundus* | Japan. Nigata Pref. | MN908981 | MN935756 |
| FLO2 | *Phedimus aizoon* var. *floribundus* | Japan. Kagawa Pref. | MN908982 | MN935757 |
| FLO3 | *Phedimus aizoon* var. *floribundus* | Japan. Osaka Pref. | MN908983 | MN935759 |
| FLO4 | *Phedimus aizoon* var. *floribundus* | Japan. Nigata Pref. | MN908984 | MN935760 |
| LAV1 | *Phedimus latiovalifolium* | Korea. Daejon | JQ954564 | - |
| TAK13 | *Phedimus takesimensis* | Korea. Ulleung-gun | MN908990 | MN935682 |
| TAK14 | *Phedimus takesimensis* | Korea. Ulleung-gun | MN909005 | MN935698 |
| OUT1 | *Rhodiola alsia* | China. Sichuan | KF113686 | KF113739 |
| OUT2 | *Rhodiola brevipetiolata* | China. Qinghai | KF113683 | KF113736 |
