# Supplementary material for: ﻿Phedimusdaeamensis (Crassulaceae), a new species from Mt. Daeam in Korea
Source: PhytoKeys. 2022 Nov 3;212:57–71. doi: 10.3897/phytokeys.212.82604 (PMC9836587; doi:10.3897/phytokeys.212.82604)
Supplement: Supplementary material 1 — Tables S1–S6 [file phytokeys-212-057_article-82604__-s001.zip › 82604_1C-1-A_revised_Table_S2 SM-1.docx]

Table S2. Diagnostic characters observed in *P. daeamensis* and the six closest related taxa.

|  | *P. aizoon* | *P. kamtschaticus* | *P. latiovalifolius* | *P. middendorffianus* | *P. takesimensis* | *P. sikokianus* | *P. daeamensis* |
| --- | --- | --- | --- | --- | --- | --- | --- |
| Habit | previous year’s flowering stem caducous or only basal parts persistent | basal part of previous year’s flowering stem persistent | previous year’s flowering stem caducous or only basal parts persistent | not seen | stems persistent | basal part of previous year’s flowering stem persistent | basal part of previous year’s flowering stem persistent |
| Root | roots tuberous; rootstock robust | roots not tuberous; rootstock not robust | roots not tuberous; rootstock not robust | roots not tuberous; rootstock not robust | roots not tuberous; rootstock not robust | roots not tuberous; rootstock not robust | roots not tuberous; rootstock not robust |
| Stems | few (1 or 2), erect, simple | numerous (3 or more), ascending with underground stolons | numerous (3 or more), erect | numerous (3 or more), tufted, creeping, erect or ascending, basally branched | numerous (3 or more), erect to ascending | numerous (3 or more), tufted, erect or ascending, basally branched | numerous (3 or more), tufted, creeping or ascending, basally branched |
| Height (flowering) | 30-46 cm | 20-43 cm | 8-15 cm | 10-30 cm | 18-30 cm | 6-15 cm | 12-21 cm |
| Leaves  -arrangement | alternate | alternate or opposite, rarely 3-verticillate | alternate | alternate | alternate | opposite | alternate |
| - petiole | sessile | sessile | sessile | sessile | sessile | sessile | sessile |
| - shape | narrowly lanceolate, elliptic-lanceolate, or ovate-oblanceolate | oblanceolate, spatulate or obovate | broadly ovate | linear-spatulate | oblanceolate | widely oblanceolate to obovate | obovate |
| - size | 3-6 cm long, 5-7 mm wide | 2.5-7 cm long, 0.5-3 cm wide | 2-4 cm long, 1.9-2.5 cm wide | 1.2-4 cm long, 0.2-0.5 cm wide | 3-4 cm long, 1.8-2.5 mm wide | 0.8-2.3 cm long, 0.6-1.3 cm wide | 1-2.3 cm long, 0.5-1.2 cm wide |
| - margins | margin crenate except near base, apex acute | margin apically sparsely serrate to crenate, apex obtuse-rounded | margins irregularly dentate, apex obtuse | margin apically serrate 2-3, apex obtuse | margin apically to mid crenate, apex obtuse | margin apically to mid crenate 2-4, apex rounded | margin apically to mid serrate 4-5, apex obtuse |
| Flowering period | May to June | June to July | May to June | June to August | June | June to July | May to June |
| Calyx lobes | 5, linear, unequal, 3-5 mm long, apex obtuse | 5, lanceolate, 3-4 mm long, apex obtuse | 5, lanceolate, 3-4 mm long, apex obtuse | 5, linear, 2-3 mm long, apex obtuse | 5, lanceolate, 2-3 mm long, apex obtuse | 5, lanceolate, 2-3 mm long, apex obtuse | 5, lanceolate, 3-4 mm long, apex obtuse |
| Petals | 5, yellow, oblong to elliptic-lanceolate, 6-10 mm long, apex long acuminate or mucronate | 5, yellow, lanceolate, 6-8 mm long, apex acuminate and mucronate | 5, yellow, lanceolate, 4-8 mm long, apex acuminate and mucronate | 5, yellow, lanceolate to linear-lanceolate, 5-11 mm long, apex acuminate and mucronate | 5, yellow, lanceolate, 5-8 mm long, apex acuminate and mucronate | 5, yellow, linear-lanceolate, 7-9 mm long, apex acuminate | 5, yellow, lanceolate to oblong, apex acuminate |
| Stamens | 10, shorter than petals; anther yellow | 10, slightly shorter than petals; anther red before dehiscence | 10, slightly shorter than petals; anther yellow | 10, shorter than petals; filaments yellow; anthers purple | 10, shorter than petals; anther red before dehiscence | 10, shorter than petals; filaments yellow; anthers reddish orange before dehiscence | 10, shorter than petals; filaments yellow; anther red |
| Carpels | ovate, 5, d at base | equaling or slightly shorter than petals, 5, briefly connate at base | shorter than petals, 5, connate at base | lanceolate, 5, briefly connate at base | lanceolate, 5, briefly connate at base | lanceolate, 5, 4.5-6 mm long, briefly connate at base | lanceolate, 5, briefly connate at base |
| Seeds | ellipsoid, ca. 1 mm long | obovoid | ellipsoid | obovoid | ellipsoid, ca. 1 mm long | ellipsoid, ca. 0.8-1 mm long | ca. 0.7-1 mm, obovoid |
