# Supplementary material for: ﻿Phedimusdaeamensis (Crassulaceae), a new species from Mt. Daeam in Korea
Source: PhytoKeys. 2022 Nov 3;212:57–71. doi: 10.3897/phytokeys.212.82604 (PMC9836587; doi:10.3897/phytokeys.212.82604)
Supplement: Supplementary material 1 — Tables S1–S6 [file phytokeys-212-057_article-82604__-s001.zip › 82604_1C-1-A_revised_Table_S3 SM-1.docx]

Table S3. Voucher information and GenBank accession numbers for 16 accessions of *Phedimus daeamensis* and closely related taxa examined in this study. All vouchers are deposited in KH unless otherwise cited.

| Accession | Locality | | | GenBank accession  number | |
| --- | --- | --- | --- | --- | --- |
|  |  |  |  | nrITS | *psb*A-*trn*H IGS |
| *Phedimus aizoon* var. *aizoon* | | | | | |
| AIZ13 | Korea, Gangwon-do, Samcheok | | | ON887309 | ON886117 |
| *Phedimus aizoon* var. *latifolius* | | | | | |
| LAT7 | Korea, Gyeongsangbuk-do, Milyang | | | ON887310 | ON886120 |
| LAT8 | Korea, Jeollanam-do, Shinan-gun | | | ON887311 | ON886121 |
| LAT10 | Korea, Gyeongsangbuk-do, Uljin-gun | | | ON887312 | ON886122 |
| *Phedimus kamtschaticus* | | | | | |
| KAM4 | Korea, Gangwon-do, Inje-gun | | | ON887316 | ON886119 |
| KAM9 | Korea, Daegu-si | | | ON887317 | ON886118 |
| *Phedimus sikokianus* | | | | | |
| SIKS8^*^ | Japan, Pref. Kochi, Kamegamori, Hongawa-mura | | | ON887319 | ON886128 |
| SIKS9^*^ | Japan, Pref. Kochi, Monobe-cho, Kami-shi | | | ON887320 | ON886129 |
| SIKS10^*^ | Japan, Pref. Kochi, Monobe-cho | | | ON887321 | ON886130 |
| SIKS11^*^ | Japan, Pref. Kochi, Nakanokawa, Ino-cho | | | ON887322 | ON886131 |
| SIKS12^**^ | Japan, Pref. Kochi, Terakawa, Ino-cho | | | ON887323 | ON886132 |
| *Phedimus middendorfianus* | | | | | |
| MID11 | China, Jilin | | | ON887318 | ON886123 |
| *Phedimus takesimensis* | | | | | |
| TAK12 | Korea, Gyeongsangbuk-do, Ulleung-gun | | | ON887324 | ON886127 |
| *Phedimus daeamensis* | |  |  |  |  |
| DAE1 | Korea, Gangwon-do, Inje-gun | | | ON887313 | ON886124 |
| DAE2 | Korea, Gangwon-do, Inje-gun | | | ON887314 | ON886125 |
| DAE3 | Korea, Gangwon-do, Inje-gun | | | ON887315 | ON886126 |

*Deposited in MBK

**Deposited in NGU
