# Supplementary material for: ﻿Phedimusdaeamensis (Crassulaceae), a new species from Mt. Daeam in Korea
Source: PhytoKeys. 2022 Nov 3;212:57–71. doi: 10.3897/phytokeys.212.82604 (PMC9836587; doi:10.3897/phytokeys.212.82604)
Supplement: Supplementary material 3 — Figure S2 [file phytokeys-212-057_article-82604__-s003.pdf]

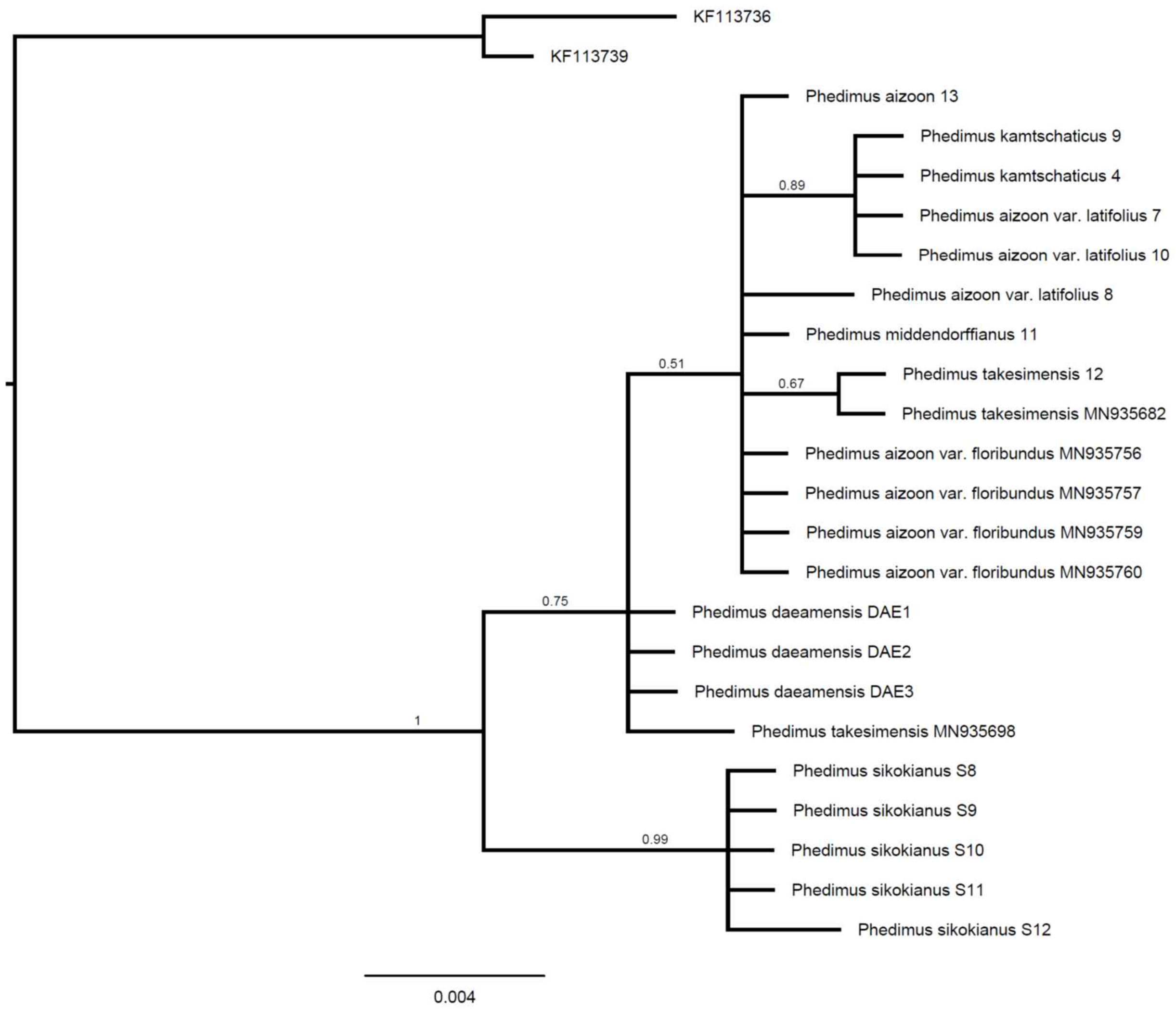

Figure S2. Bayesian inference tree for individuals of *P. daeamensis* and related taxa based on *psb*A-*trn*H IGS. Numbers above branches are posterior probabilities
